# Supplementary material for: Associating Biological Activity and Predicted Structure of Antimicrobial Peptides from Amphibians and Insects
Source: Antibiotics (Basel). 2022 Nov 27;11(12):1710. doi: 10.3390/antibiotics11121710 (PMC9774241; doi:10.3390/antibiotics11121710)
Supplement: Supplementary file 1 [file antibiotics-11-01710-s001.zip › antibiotics-2036844-supplementary.pdf]

Supplementary material for:

Associating biological activity and predicted structure of antimicrobial peptides from amphibians and insects

Amelia Richter, Darcy Sutherland, Hossein Ebrahimikondori, Alana Babcock, Nathan Louie, Chenkai Li, Lauren Coombe, Diana Lin, René L. Warren, Anat Yanai, Monica Kotkoff, Caren C. Helbing, Fraser Hof, Linda M.N. Hoang, Inanc Birol

Additional File 1

Table of Contents

Table S1. Minimum inhibitory concentrations of peptides from three independent experiments 2

Table S2. Highest Scoring BLASTp matches of validated mature AMPs to NCBI non-redundant protein database 5

Table S3. Range of selectivity indices of the 51 bioactive AMPs 8

Figure S1. Superimposed predicted 3D structures of AMP family members 10

Supplementary Tables

Table S1. Minimum inhibitory concentrations of peptides from three independent experiments

Peptide		MIC (µg/mL)		
		E. coli **ATCC 25922**	***Salmonella*** **Enteritidis**	***S. aureus*** **ATCC 29213**
GILDTLKQLGKAAVQGLLSKAACKLAKTC	AmMa1	2-4	16-32	8
DNKWQNVHFHRSAVTGPTSFSFSHK	AnFl1	>128	>128	>128
GILRSLGWIQMPRSRRRHR	AnFl2	16-32	128	>128
GIYTGRLLPVYIPQPRPPHPRLRR	ApCe1	4-8	16-32	>128
VKCRVRR	ApMe1	>128	>128	>128
GAHKEVFKRDTALTKEAAKKAKK	ApMe2	>128	>128	>128
GWGLINIKIPPVLHKVSVPLVSKR	ApMe3	>128	>128	>128
KHHHIKLRHERHRRYILKSLI	ApMe4	>128	>128	>128
SILSTLSHKR	ApMe5	>128	>128	>128
RARKIRRRRGSLRHCVTIPSTPSGR	ApMe6	>128	>128	>128
AAGAGKVTKSAQKAQKAK	BoAr1	>128	>128	>128
ATAAECLKHPWLKIKK	BoAr2	>128	>128	>128
IIRATAAECLKHPWLKIKK	BoAr3	>128	>128	>128
SVASLAKNSAWPVSLKR	BoAr4	>128	>128	>128
VTISIARRVSSHKRG	BoAr5	>128	>128	>128
GILRLVTRRFRFSPTNLNRYTVARLVSGVP	BoAr6	32	64	64-128
NKIKFINKYVKKVQLKKILVKS	BoCo1	>128	>128	>128
RKIIAVSVHKLCRVKR	BoUs1	64-128	64	>128
FACPIGFFRLKR	CaCa1	128	>128	>128
FIKTQVLKHLVAGVRVARGLDWKWR	CaCa2	8	>128	64-128
KHHHIKLRHGRHRRSVLRTLV	CaCa3	>128	>128	>128
RRFFFATAPCGYSRKFCKITRRKR	CaCa4	16-32	32-64	>128
GAFVLWGPTPRPRRR	DiLo1	128	>128	>128
GIFSLIKGAAK	LeBo1	>128	>128	>128
GWLDIAKKVASVVAGIVKR	LiVe1	4-8	32	16-32
GWLDIAKKVASVVAGLGKR	LiVe2	4-8	64-128	64
VMLPKFKR	MiDe1	>128	>128	>128
RRAIFASIRGYLGLRKR	MyGu1	4	8-16	128
TPLSDIFRGQLRSRVSR	NaVi1	>128	>128	>128
SSLSPLSSSSGLGKKKKRKSKRASR	NaVi2	>128	>128	>128

KLFLTLWKLKR	NaVi3	8-16	64	128
GSSSRSCRCIRLSRLSSKRT	NaVi4	>128	>128	>128
GLLSGILGAGKKIVCGFSGLC	OdMa1	8-16	128	64
GLISGILGAGKKVLCGLSGLC	OdMa10	16-32	>128	64-128
GFMDTAKNVAKNVAVTLLYNLKCKITKAC	OdMa12	4	64	64-128
GFMDTAKNVAKNVAVTLLDNLKCKITKAC	OdMa13	64-128	>128	>128
GLLRGILGAGKKIVCGLSGLC	OdMa2	4	8-16	4-8
GLLSGLLGAGKKIVCGLSGMC	OdMa3	8-16	64	32
GILSGLLGAGKKIVC	OdMa4	8-16	32	>128
GILSGLLGAGKKIVCGLSGLC	OdMa5	8-16	64-128	8-16
GLLSGVLGVGKKIVCGLSGLC	OdMa6	8-16	128	16-32
GLLSGVLGVGKKVLCGLSGLC	OdMa7	8-16	128	16-32
GLISGILGAGKK	OdMa8	>128	>128	>128
GLISGILGAGKKVLC	OdMa9	64-128	128	>128
GILSGLLGAGKKLACGLIGLC	OdTo1	8	32-64	8-16
GIFGGHLKVGKKIACGLSGLC	OdTo2	32	>128	>128
GIFGGLLKEGKKIACGLSGLC	OdTo3	32	>128	>128
KLMIPRKKRGIFGGLLKVGKKIACGLSGLC	OdTo4	8-16	16	16
RPRPQQVPPRPPHPRLRR	PaVa1	2-4	4-8	>128
KYHHIKLRHGRHRRTIH	PaVa2	>128	>128	>128
ITEPVGTKAPTFTSELRGGWLKKR	PaVa3	>128	>128	>128
WALRWKTR	PaVi1	>128	>128	>128
GLLGKVLGVGKKVLCGVTGLC	PeNi1	4	8-16	4
GLLLDTVKGAAKNVAGILLNKLKCKVTGDC	PeNi10	8	64-128	16-32
GILTDTLKGAAKNVAGVLLDKLKCKITGGC	PeNi11	8-16	128	64
GAPKGCWTKSYPPKPCSGKR	PeNi12	>128	>128	>128
KEERGAPKGCWTKSYPPKPCSGKR	PeNi13	>128	>128	>128
GLWTTIKEGVKNFSVGVLDKIRCKITGGC	PeNi14	4-8	32	16-32
FLPSSPWNEGTYVLKKLKS	PeNi15	>128	>128	>128
ATAWKVPPGLQPIRPIRIRPLCGNDKS	PeNi16	>128	>128	>128
RMIRRPPGFSPFRVAPASSLKR	PeNi17	>128	>128	>128
RPRWSHRSRR	PeNi18	>128	>128	>128
GLLGKVLGVGKKVLCVVSGLC	PeNi2	4	16-32	4
GIFSLIKGAAKVVAKGLG	PeNi3	16-32	64-128	64-128
GLLGKVLGVGKKVLC	PeNi4	8-16	4-8	4-8
GLLGKVLGVGKKVLCGVTGRERCQ	PeNi5	32	64	>128
AGLQFPVGRIHRHLKTR	PeNi6	>128	>128	>128
VIPFVASVAAEMMHHVYCAASKRCKN	PeNi7	128	>128	>128
GILLNTLKGAAKNVAGVLLDKLKCKITGGC	PeNi8	8	64	4-8

GLLGKILGVGKKVLCGVSGLC	PeNi9	2-4	8-16	4-8
VAAFAIIGCLCCRRPRR	PoRo1	>128	>128	>128
ISIKEALEHSFFHTVPRKWCKKH	PoSn1	64	>128	>128
TALKSLSILKKLAKLNM	PoSn2	64-128	>128	>128
FLPVVAGLAAKVLPSIICAVTKKC	RaCa15	8-16	>128	4-8
GLLSGILGAGKK	RaOm1	>128	>128	>128
GILSGLLGAGKKIVCGLSGMC	RaOm2	64-128	>128	>128
GIFSLIKGAAKVVAKGLGK	RaOm3	8-16	32-64	64
GLLGKVLGVGKKVLCGVSGRC	RaOm4	4-8	32	64-128
AGYSRMIRRPPGFSPFRVAPASSLKR	RaOm5	>128	>128	>128
GLVGKLVKGGLKLIGHVANG	RaSi1	8-16	>128	64-128
FPFPFGRR	RaSi2	>128	>128	>128
EEQRFLPVVAGLAAKVLPSIICAVTKKC	RaSy2	64	>128	64-128
KIKIPWGKVKDFLVGGMKAVGKK	TeBi1	1-2	4-8	4-8
VPFGLKPR	TeRu1	>128	>128	>128
AFVRILCYCCPRRIKRR	TeRu2	64-128	>128	128
AVLSFVHKLFLNFLHVDTSKGKCRATLQ	TeRu3	>128	>128	>128
SWLSKSVKKLVNKKNYTRLEKLAKKKLFNE	TeRu4	1	2-4	>128
FILHAKKTRSAK	VeSi1	>128	>128	>128

Table S2. Highest Scoring BLASTp matches of validated mature AMPs to NCBI non-redundant protein database

Peptide		BLASTp Results				
**ID**	**Source Organism**	**Accession**	**Label**	**Organism**	**% Identity**	**% Query Coverage**
AmMa1	*Amolops mantzorum*	ADM34231.1	palustrin-2GN3 antimicrobial peptide precursor	*Amolops granulosus*	93	100
AnFl2	*Anterhynchium flavomarginatum*	NA				
ApCe1	*Apis cerana*	NA				
BoAr6	*Bombus ardens*	NA				
BoUs1	*Bombus ussurensis*	NA				
CaCa1	*Camponotus castaneus, Odontomachus monticola, Polistes rothneyi, Polistes snelleni, Sphecidae sp. KJ-8906, Vespa dybowskii*	XP_044595060.1	uncharacterized protein LOC123272390	*Cotesia glomerata*	100	100
CaCa2	*Camponotus castaneus, Odontomachus monticola, Temnothorax rugatulus*	XP_032682614.1	E3 ubiquitin-protein ligase HECTD1 isoform X1	*Odontomachus brunneus*	100	100
CaCa4	*Camponotus castaneus*	XP_012226981.1	PREDICTED: F-box/LRR-repeat protein 20 isoform X1	*Linepithema humile*	95	92
DiLo	*Diachasmimorpha longicaudata*	XP_008546965.1	PREDICTED: ubiquitin carboxyl-terminal hydrolase 30 homolog	*Microplitis demolitor*	93	100
LiVe1	*Litoria verreauxii*	NA				
LiVe2	*Litoria verreauxii*	NA				
MyGu1	*Myrmecia gulosa*	NA				
NaVi3	*Nasonia vitripennis x Nasonia giraulti F1*	NA				
OdMa1	*Odorrana margaretae*	AWH66435.1	nigrocin-HL precursor	*Hylarana latouchii*	95	100
OdMa2	*Odorrana margaretae*	ABG76398.1	nigrosin-OG15 antimicrobial peptide precursor	*Odorrana grahami*	95	100
OdMa3	*Odorrana margaretae*	AWH66435.1	nigrocin-HL precursor	*Hylarana latouchii*	90	100
OdMa4	*Odorrana margaretae*	NA				
OdMa5	*Odorrana margaretae*	ACA81697.1	lividin-7b precursor	*Odorrana livida*	100	100
OdMa6	*Odorrana margaretae*	ADP05878.1	nigrosin-RA3 peptide precursor	*Odorrana andersonii*	100	100
OdMa7	*Odorrana margaretae*	ABG76389.1	nigrosin-OG21 antimicrobial peptide precursor	*Odorrana grahami*	100	100
OdMa9	*Odorrana margaretae*	NA				
OdMa10	*Odorrana margaretae*	ADM34194.1	nigrosin-MG1 antimicrobial peptide precursor	*Odorrana margaretae*	100	100
OdMa12	*Odorrana margaretae*	ABG76517.1	odorranain-F2 antimicrobial peptide precursor	*Odorrana grahami*	97	100
OdMa13	*Odorrana margaretae*	ABG76517.1	odorranain-F2 antimicrobial peptide precursor	*Odorrana grahami*	100	100
OdTo1	*Odorrana tormota*	ADE48808.1	nigrosin-SHa antimicrobial protein	*Odorrana schmackeri*	81	100

OdTo2	*Odorrana tormota*	AIU99961.1	nigrosin-2EV4	*Odorrana exiliversabilis*	71	100
OdTo3	*Odorrana tormota*	AIU99958.1	nigrosin-2EV1	*Odorrana exiliversabilis*	76	100
OdTo4	*Odorrana tormota*	AIU99958.1	nigrosin-2EV1	*Odorrana exiliversabilis*	78	77
PaVa1	*Partula varia*	XP_024940855.1	apidaecins type 14-like isoform X2	*Cephus cinctus*	100	100
PeNi1	*Pelophylax nigromaculatus*	AEM68196.1	nigrocin-2N protein precursor, partial	*Pelophylax nigromaculatus*	95	100
PeNi2	*Pelophylax nigromaculatus*	AEM68196.1	nigrocin-2N protein precursor, partial	*Pelophylax nigromaculatus*	95	100
PeNi3	*Pelophylax nigromaculatus*	AEM68180.1	esculentin-2N protein precursor, partial	*Pelophylax nigromaculatus*	100	100
PeNi4	*Pelophylax nigromaculatus*	NA				
PeNi5	*Pelophylax nigromaculatus*	AEM68196.1	nigrocin-2N protein precursor, partial	*Pelophylax nigromaculatus*	95	79
PeNi7	*Bufo gargarizans, Leptobrachium boringii, Megophrys sangzhiensis, Polypedates megacephalus, Pelophylax nigromaculatus, Rhacophorus dennysi, Rana omeimontis*	AEM68212.1	nigrocin-6N protein precursor, partial	*Pelophylax nigromaculatus*	100	100
PeNi8	*Bufo gargarizans, Megophrys sangzhiensis, Polypedates megacephalus, Pelophylax nigromaculatus, Rhacophorus dennysi, Rana omeimontis*	AGE10575.1	pelophylaxin-2	*Pelophylax nigromaculatus*	100	100
PeNi9	*Leptobrachium boringii, Megophrys sangzhiensis, Polypedates megacephalus, Pelophylax nigromaculatus, Rhacophorus dennysi, Rana omeimontis*	AEM68196.1	nigrocin-2N protein precursor, partial	*Pelophylax nigromaculatus*	95	100
PeNi10	*Leptobrachium boringii, Polypedates megacephalus, Pelophylax nigromaculatus, Rhacophorus dennysi, Rana omeimontis*	AEM68233.1	ranatuerin-2N protein precursor, partial	*Pelophylax nigromaculatus*	100	100
PeNi11	*Leptobrachium boringii, Polypedates megacephalus, Pelophylax nigromaculatus, Rhacophorus dennysi, Rana omeimontis*	AIU99901.1	pelophylaxin-1 antimicrobial peptide precursor	*Pelophylax hubeiensis*	100	100
PeNi14	*Bufo gargarizans, Polypedates megacephalus, Pelophylax nigromaculatus, Rana omeimontis*	AIU99897.1	palustrin-2HB1	*Pelophylax hubeiensis*	86	100
PoSn1	*Polistes snelleni*	EFN63611.1	Phosphorylase b kinase gamma catalytic chain, skeletal muscle isoform	*Camponotus floridanus*	93	65
PoSn2	*Polistes snelleni*	NA				
RaCa15	*Rana catesbeiana*	P82871.1	RecName: Full=Brevinin-1SY	*Lithobates sylvaticus*	100	100
RaOm2	*Rana omeimontis*	ADE48808.1	nigrosin-SHa antimicrobial protein	*Odorrana schmackeri*	90	100
RaOm3	*Rana omeimontis*	AEM68180.1	esculentin-2N protein precursor, partial	*Pelophylax nigromaculatus*	100	100

RaOm4	*Rana omeimontis*	AEM68196.1	nigrocin-2N protein precursor, partial	*Pelophylax nigromaculatus*	95	100
RaSi1	*Allobates femoralis, Pristimantis toftae, Ranitomeya sirensis*	NA				
RaSy2	*Rana sylvatica*	ABK91541.1	brevinin-1CHb precursor, partial	*Lithobates chiricahuensis*	78	96
TeBi1	*Tetramorium bicarinatum*	W8GNV3.1	Full=M-myrmicitoxin(01)-Tb1a; Short=M-MYRTX(01)-Tb1a	*Tetramorium bicarinatum*	100	100
TeRu2	*Temnothorax rugatulus*	XP_020294524.1	dopamine receptor 2 isoform X1	*Pseudomyrmex gracilis*	94	100
TeRu4	*Temnothorax rugatulus*	XP_024884948.1	uncharacterized protein LOC112463023	*Temnothorax curvispinosus*	97	100

Table S3. Range of selectivity indices of the 51 bioactive AMPs

AMP	Selectivity Index		
	***E. coli* ATCC 25922**	***Salmonella* Enteritidis**	***S. aureus* ATCC 29213**
AmMa1	32 - >64	≥16	4 - >8
AnFl2	>4 - >8	N/O*	>1
ApCe1	>16 - >32	N/O	>4 - >8
BoAr6	>4	>1 - >2	>2
BoUs1	>1 - >2	N/O	>2
CaCa1	>1	N/O	N/O
CaCa2	>16	>1 - >2	N/O
CaCa4	>4 - >8	N/O	>2 - >4
DiLo1	>1	N/O	N/O
LiVe1	>16 - >32	>4 - >8	>4
LiVe2	>16 - >32	>2	>1 - >2
MyGu1	>32	>1	>8 - >16
NaVi3	>8 - >16	>1	>2
OdMa1	>8 - >16	>2 - >4	>1
OdMa2	32	16 - 32	8 - 16
OdMa3	>8 - >16	>4	>2
OdMa4	>8 - >16	N/O	>4
OdMa5	8 - 16	8 - 16	1 - 2
OdMa6	>8 - >16	>4 - >8	>1
OdMa7	>8 - >16	>4 - >8	>1
OdMa9	>1 - >2	N/O	>1
OdMa10	>4	>1 - >2	N/O
OdMa12	32	>1 - >2	>2
OdMa13	>1 - >2	N/O	N/O
OdTo1	≥16	8 - >16	2 - >4
OdTo2	>4	N/O	N/O
OdTo3	>2 - >4	N/O	N/O
OdTo4	>8 - >16	>8	>8
PaVa1	>32 - >64	N/O	>16 - >32
PeNi1	≥32	≥32	8 - >16
PeNi2	>32	>32	>4 - >8
PeNi3	>4 - >8	>1 - >2	>1 - >2
PeNi4	>8 - >16	>16 - >32	>16 - >32
PeNi5	>4	N/O	>2
PeNi7	>1	N/O	N/O
PeNi8	>16	>16 - >32	>2

PeNi9	32 - >64	16 - >32	8 - >16
PeNi10	>16	>4 - >8	>1 - >2
PeNi11	>8 - >16	>2 - >4	>1
PeNi14	>16 - >32	>4 - >8	>4
PoSn1	>2	N/O	N/O
PoSn2	>1 - >2	N/O	N/O
RaCa15	4 - 16	8 - 32	N/O
RaOm2	>1 - >2	N/O	N/O
RaOm3	>8 - >16	>2	>2 - >4
RaOm4	>16 - >32	>1 - >2	>4
RaSi1	>8 - >16	>1 - >2	N/O
RaSy2	>2	>1 - >2	N/O
TeBi1	>64 - >128	>16 - >32	>16 - >32
TeRu2	>1 - >2	N/O	N/O
TeRu4	>128	N/O	>32 - >64

*N/O indicates no observed antimicrobial activity against the given bacterial species

Supplementary Figures

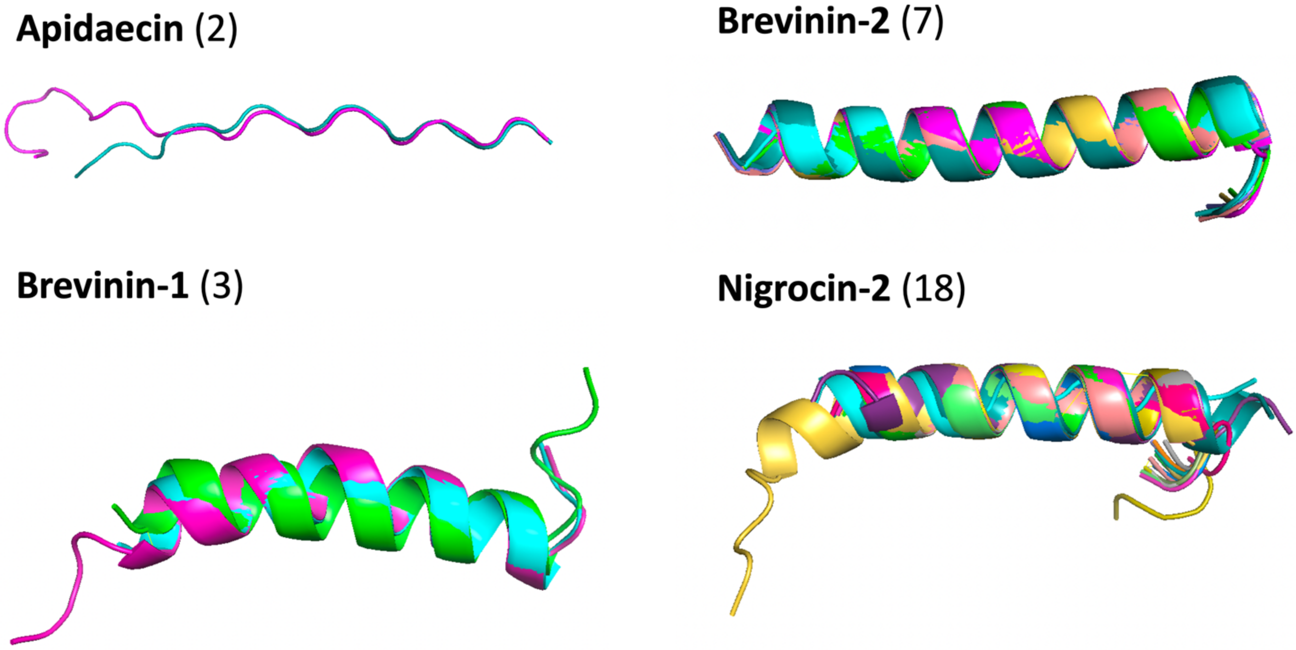

Figure S1. Superimposed predicted 3D structures of AMP family members. Peptides identified as belonging to the Apidaecin, Brevinin-1, Brevinin-2 and Nigrocin-2 peptides within the 51 bioactive peptides are depicted.
